# Identification and analysis of prognostic immune cell homeostasis characteristics in lung adenocarcinoma

**DOI:** 10.1111/crj.13755

**Published:** 2024-05-17

**Authors:** Yidan Sun, Qianqian Ma, Yixun Chen, Dongying Liao, Fanming Kong

**Affiliations:** ^1^ Department of Oncology First Teaching Hospital of Tianjin University of Traditional Chinese Medicine Tianjin China; ^2^ Affiliated Women's Hospital of Jiangnan University Wuxi Jiangsu China; ^3^ Research Center of Clinical Medicine Affiliated Hospital of Nantong University Nantong Jiangsu China

**Keywords:** gene signature, immune homeostasis, lung adenocarcinoma, prognosis, TCGA

## Abstract

**Background:**

Lung adenocarcinoma (LUAD) is one of the most invasive malignant tumor of the respiratory system. It is also the common pathological type leading to the death of LUAD. Maintaining the homeostasis of immune cells is an important way for anti‐tumor immunotherapy. However, the biological significance of maintaining immune homeostasis and immune therapeutic effect has not been well studied.

**Methods:**

We constructed a diagnostic and prognostic model for LUAD based on B and T cells homeostasis‐related genes. Minimum absolute contraction and selection operator (LASSO) analysis and multivariate Cox regression are used to identify the prognostic gene signatures. Based on the overall survival time and survival status of LUAD patients, a 10‐gene prognostic model composed of ABL1, BAK1, IKBKB, PPP2R3C, CCNB2, CORO1A, FADD, P2RX7, TNFSF14, and ZC3H8 was subsequently identified as prognostic markers from The Cancer Genome Atlas (TCGA)‐LUAD to develop a prognostic signature. This study constructed a gene prognosis model based on gene expression profiles and corresponding survival information through survival analysis, as well as 1‐year, 3‐year, and 5‐year ROC curve analysis. Enrichment analysis attempted to reveal the potential mechanism of action and molecular pathway of prognostic genes. The CIBERSORT algorithm calculated the infiltration degree of 22 immune cells in each sample and compared the difference of immune cell infiltration between high‐risk group and low‐risk group. At the cellular level, PCR and CKK8 experiments were used to verify the differences in the expression of the constructed 10‐gene model and its effects on cell viability, respectively. The experimental results supported the significant biological significance and potential application value of the molecular model in the prognosis of lung cancer. Enrichment analyses showed that these genes were mainly related to lymphocyte homeostasis.

**Conclusion:**

We identified a novel immune cell homeostasis prognostic signature. Targeting these immune cell homeostasis prognostic genes may be an alternative for LUAD treatment. The reliability of the prediction model was confirmed at bioinformatics level, cellular level, and gene level.

## Graphical abstract

Despite the presence of tumor markers in LUAD, studies based on genes associated with maintaining immune homeostasis have not been reported. We constructed a prognostic model of LUAD based on immune cell homeostasis‐related genes. LASSO and multivariate Cox regression were used to identify prognostic genes. Based on the overall survival time and survival status of TCGA‐LUAD patients, 10 gene prognostic models consisting of ABL1, BAK1, IKBKB, PPP2R3C, CCNB2, CORO1A, FADD, P2RX7, TNFSF14, and ZC3H8 were identified as prognostic markers.

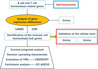

## 1 INTRODUCTION

Lung adenocarcinoma (LUAD) has a relatively poor prognosis and therefore requires better clinical prognostic predictors and therapeutic strategies.^1^ Negative feedback regulation of immunohomeostasis maintenance plays an important role in tumor.^2^, ^3^ Immunoresponse markers have prognostic and predictive effects in patients with lung cancer. At the same time, with the rapid development of network genome research, bioinformatics analysis based on tumor clinical data in The Cancer Genome Atlas (TCGA) has made up for the shortage of clinical data collection difficulties, and it is feasible to establish an accurate stratified survival risk model of LUAD patients based on gene markers. Negative feedback regulation of immune homeostasis plays an important role in cancer.

The balance mechanism between tumor and immune infiltrating cells maintains a problematic immune microenvironment and pathogenesis of tumors. in tumor pathogenesis.^4^ This is mainly due to failure of cytotoxic lymphocyte deployment or suppression of immunosuppressive checkpoints.^5^ Studies reported key gene receptors are differentially expressed during immune cell activation to ensure the balance of immune homeostasis.^6^ The activity of genes required for recruitment of immune cells was higher, and the activity of cytoplasmic nucleic acid sensing pathway was lower. The gene set associated with immune homeostasis may be associated with enhanced immunogenicity during tumor development.^7^ The applicability of these key prognostic gene targeting pathways and their changes in tumor microenvironment (TME) factors may be potential targets for regulating cancer signaling pathways.

In this study, we used gene expression data from publicly available resources in TCGA to construct a correlation network of immunohomeostasis‐related genes and to define gene clusters closely related to LUAD. At the same time, RNAseq analysis in BEAS‐2B, H1975, and H1395 cell lines revealed differences in the expression of key genes in LUAD. It was further found that the cell activity decreased after inhibiting FADD expression. On the contrary, inhibition of CORO1A expression increased the cell activity. The reliability of the prediction model was confirmed at bioinformatics level, cellular level, and gene level.

## 2 MATERIALS AND METHODS

### 2.1 Data collection

Tertiary data for mRNA sequencing in LUAD were obtained from TCGA. The downloaded gene expression data are in the form of “One million fragments per kilobase” (FPKM). The raw data are then converted to a “Transcription number in millionths” (TPM).

### 2.2 Identification of DE‐immnue cell homeostasis genes

We retrieved the “B Cell Homeostasis” and “T Cell Homeostasis” gene sets in the Harmonizome (https://maayanlab.cloud/Harmonizome/) database with the search term “Immune Cell Homeostasis.” The 41 non‐duplicated genes in the two sets were synthesized as immune cell homeostasis‐related genes for the construction of prognostic models. Differential gene expression analysis is assessed using limma.^8^

### 2.3 Enrichment analysis

Gene Ontology (GO)^9^, ^10^ and Kyoto Encyclopedia of Genes and Genomes (KEGG) analyses were performed using relevant software packages with an accepted threshold.^11^, ^12^

### 2.4 Machine learning selection of biomarkers

To construct a model with perfect predictive performance, we used machine learning models to select genes significantly associated with prognosis. The gene expression values were normalized by “Minmax” normalization methods. The least absolute shrinkage and selection operator (LASSO) algorithm was used for further analysis of prognostic associated hub gene for dimensional reduction analysis and feature selection.^13^

$$\text{risk}\text{Score}=\sum_{i}\text{Coefficient}\;\left(\mathrm{hub}\;{\text{gene}}_{i}\right)*\text{mRNA Expression}\;\left(\mathrm{hub}\;{\text{gene}}_{i}\right)$$

### 2.5 The construction and validation of risk model

The important values of genes in the two models were calculated, and the 10 genes with the most important values were selected as pivotal genes for further study. Prognostic analysis of clinical data was extracted from TCGA database. Survival analysis showed difference of overall survival (OS). In addition, Cox regression analysis and receiver operating characteristic (ROC) are used for discrimination.^14^

### 2.6 Evaluation of TME

Use CIBERSORT algorithm in R to determine the TME status and levels of infiltrating lymphocyte (TILs) for each LUAD sample.^15^, ^16^ CIBERSORT was used to predict the relative in LUAD samples. *p* value ≤0.05 was used as the selection criterion to ensure the reliability of the prediction results.^17^

### 2.7 Cell culture and transient transfection

BEAS‐2B, H1975, and H1395 cells were purchased from Beijing Bena Biotechnology Co. (Beijing, China). Cells were cultured in DEME F‐12 medium. The negative control (NC), FADD siRNA, and CORO1A siRNA (Sagon, China) were transfected into the cells utilizing Lipofectamine 2000 (Invitrogen, USA).

### 2.8 Quantitative real‐time polymerase chain reaction (qRT‐PCR)

TRIzol reagent was used to extract total RNA from BEAS‐2B, H1975, and H1395 cell lines (Thermo Fisher, USA). cDNA was produced using the HiScript II SuperMix (Vazyme, China) from 500 ng of RNA. ABI 7500 System was used to perform qRT‐PCR using the SYBR Green Master Mix. The PCR amplification conditions were 45 cycles of 94°C for 10 min,94°C for 10 s, and 60°C for 45 s each. The internal reference was GAPDH. The primer pair sequences for the genes that were being targeted are listed below:

**Table:** 

Gene	Forward primer sequence (5′‐3′)	Reverse primer sequence (5′‐3′)
TNFSF14	GGTCTCTTGCTGTTGCTGATGG	TTGACCTCGTGAGACCTTCGCT
CCNB2	CAACCAGAGCAGCACAAGTAGC	GGAGCCAACTTTTCCATCTGTAC
FADD	CTCCTGCGCGAGCTGCTCGC	GCCTTCTCCAATCTTTCCCCAC
CORO1A	CCAACATCGTCTACCTCTGTGG	CTCACACTTGTTCACCTCCAGG
ABL1	CCAGGTGTATGAGCTGCTAGAG	GTCAGAGGGATTCCACTGCCAA
GAPDH	AATGGGCAGCCGTTAGGAAA	GCCCAATACGACCAAATCAGAG

### 2.9 Cell viability

Cell viability was detected using the Cell Counting Kit‐8 assay (Beyotime, China), according to the manufacturer protocol. Cells from different treatments were cultured in 96‐well plates at a density of 1 × 10^3^ cells per well. CCK‐8 solution was applied at the indicated time points. After incubation at 37°C for 2 h, the OD 450 values of each well were detected using a microplate reader (BioTeK, USA).

### 2.10 Statistical analysis

Statistical analysis was performed using R software (v.4.0.1). Wilcoxon sign rank test was used to analyze the correlation between gene expression and clinicopathological features. To assess the effects of gene expression on survival and clinicopathological factors, Kaplan–Meier Kruskal–Wallis test was used to compare OS differences between groups. We used Cox regression to assess OS in TCGA patients. *p* < 0.05 is considered significant.

## 3 RESULTS

### 3.1 Construction of LASSO model based on immune cell homeostasis of OS in LUAD

**FIGURE 1:**
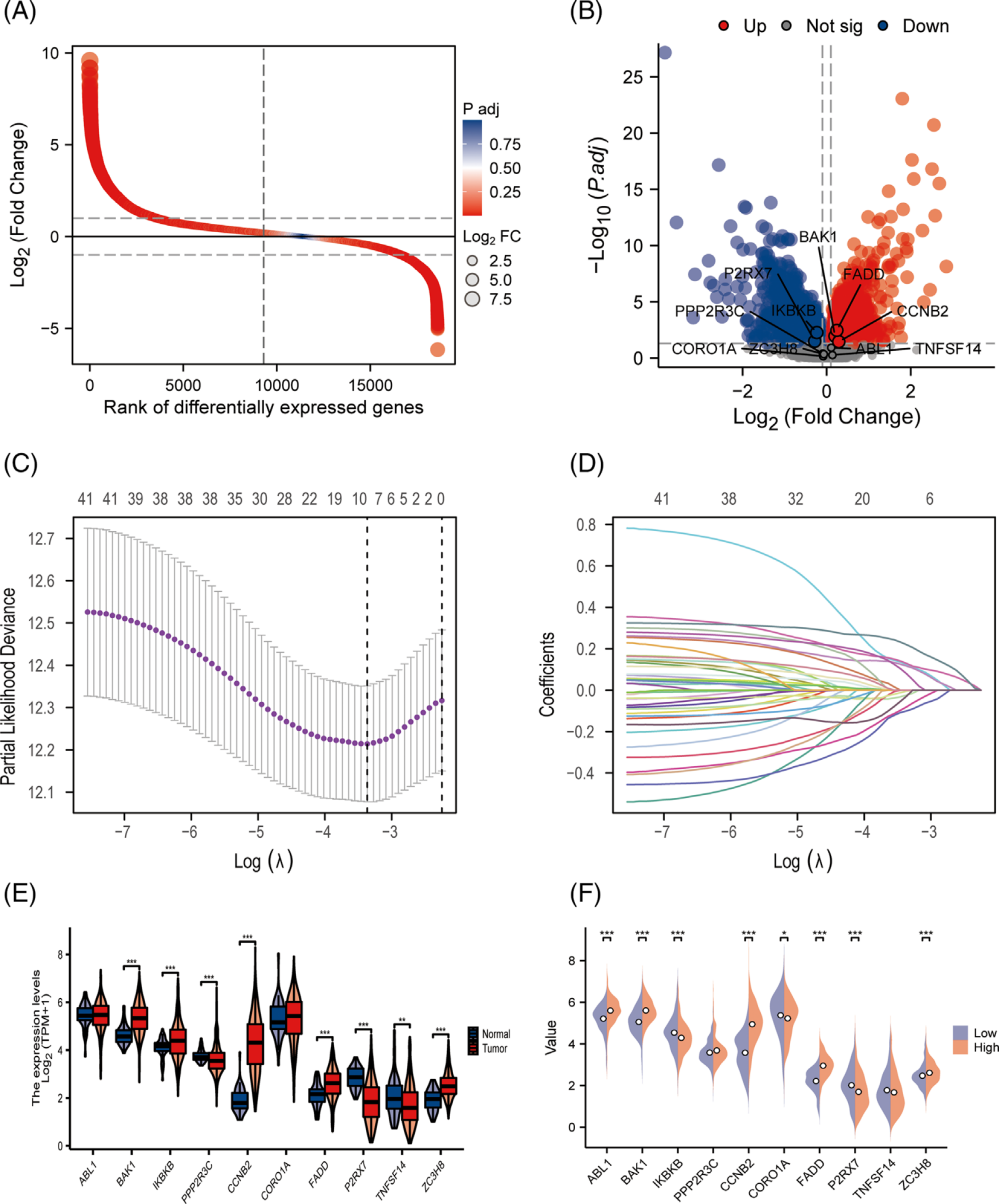
Establishment of prognostic risk scoring model. (A) Differential expression ratio curve of translation protein gene between normal and tumor tissues in TCGA‐LUAD cohort; (B) differentially expressed gene volcano map, marked significantly different immune homeostasis genes; (C) LASSO coefficients of 41 immune cell homeostasis‐associated genes; (D) genetic identification of prognostic risk score model development; (E) box plot of expression difference of risk prognostic genes in different tissues in TCGA‐LUAD database; and (F) box plot of the differential expression of prognostic risk model genes in TCGA‐LUAD database.

We first analyzed the differential expression of translation protein genes with tumor tissues in the TCGA‐LUAD and marked significantly different immune homeostasis genes by differential sequencing plot and volcano plot (Figure 1A,B).

**FIGURE 2:**
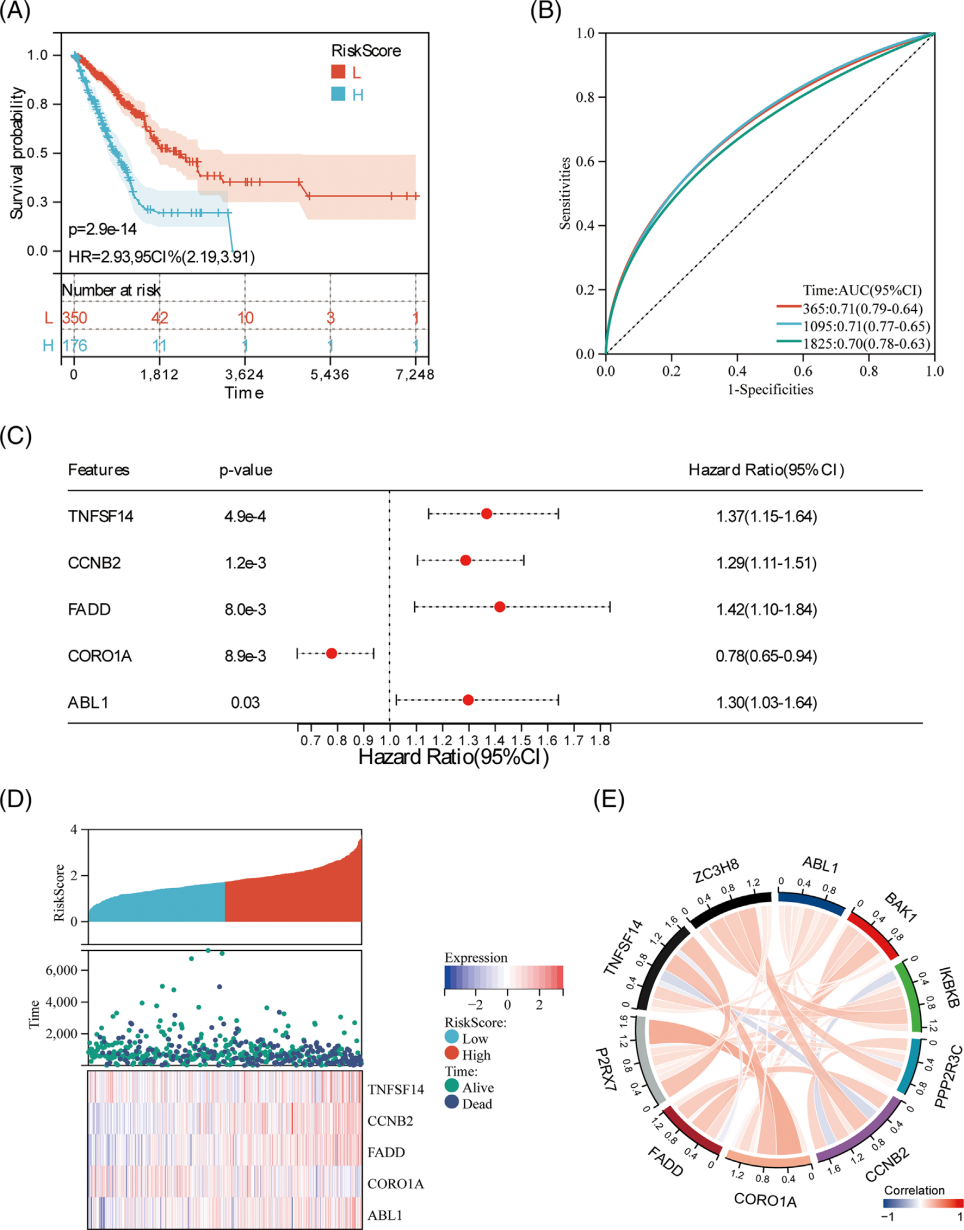
Survival prognosis analysis and intergene correlation of the risk model. (A) Survival differences in the prognostic model; (B) prognostic model risk score 1‐, 3‐, and 5‐year ROC curves; (C) forest plots of five regulatory factors with prognostic value in multivariate Cox regression models; (D) the relationship between risk scores, expression levels of risk factors and patient survival predicted by the model; and (E) chords of risk prognostic genes in TCGA‐LUAD database.

In view of the biological significance of immune cell homeostasis in the tumor process, we constructed a diagnostic and OS risk model of LUAD based on 41 genes related to immune cell homeostasis in WP database, which can be used for regression analysis of diagnosis and prognosis. First, we determined the optimal *λ* value by LASSO regression analysis, and 10 statistically significant genes were screened. A 10‐gene diagnostic model consisting of ABL1, BAK1, IKBKB, PPP2R3C, CCNB2, CORO1A, FADD, P2RX7, TNFSF14, and ZC3H8 was obtained. A prognostic risk score model based on LUAD OS was established and identified according to the LASSO coefficients of 41 immune cell homeostasis‐related genes (Figure 1C,D). Subsequently, the expression differences of risk genes in the TCGA‐LUAD cohort between different tissues and in high‐risk groups were identified (Figure 1E,F). The correlation of prognostic risk genes expressed in the TCGA‐LUAD database was demonstrated by chorography (Figure 2E). We identified significant differences in the expression of the model genes in the TCGA‐LUAD (Figure S1).

### 3.2 Survival prognosis analysis of the risk model

We analyzed the survival difference of the risk score in high‐risk samples of our risk model, and the results suggested that the high‐risk group had a significantly worse prognosis (Figure 2A). ROC was calculated to verify the ability of our risk module. We compared survival results between high‐ and low‐risk groups in the gene signature (Figure 2B).

Next, forest maps obtained by multivariate Cox regression analysis show five risk genes with prognostic value. TNFSF14, CCNB2, FADD, and ABL1 are risk factors for LUAD patients, while CORO1A is a protective factor for LUAD. The heat map showed the expression patterns of five OS and prognostic independent risk genes in patients with different risk scores (Figure 2D).

**FIGURE 3:**
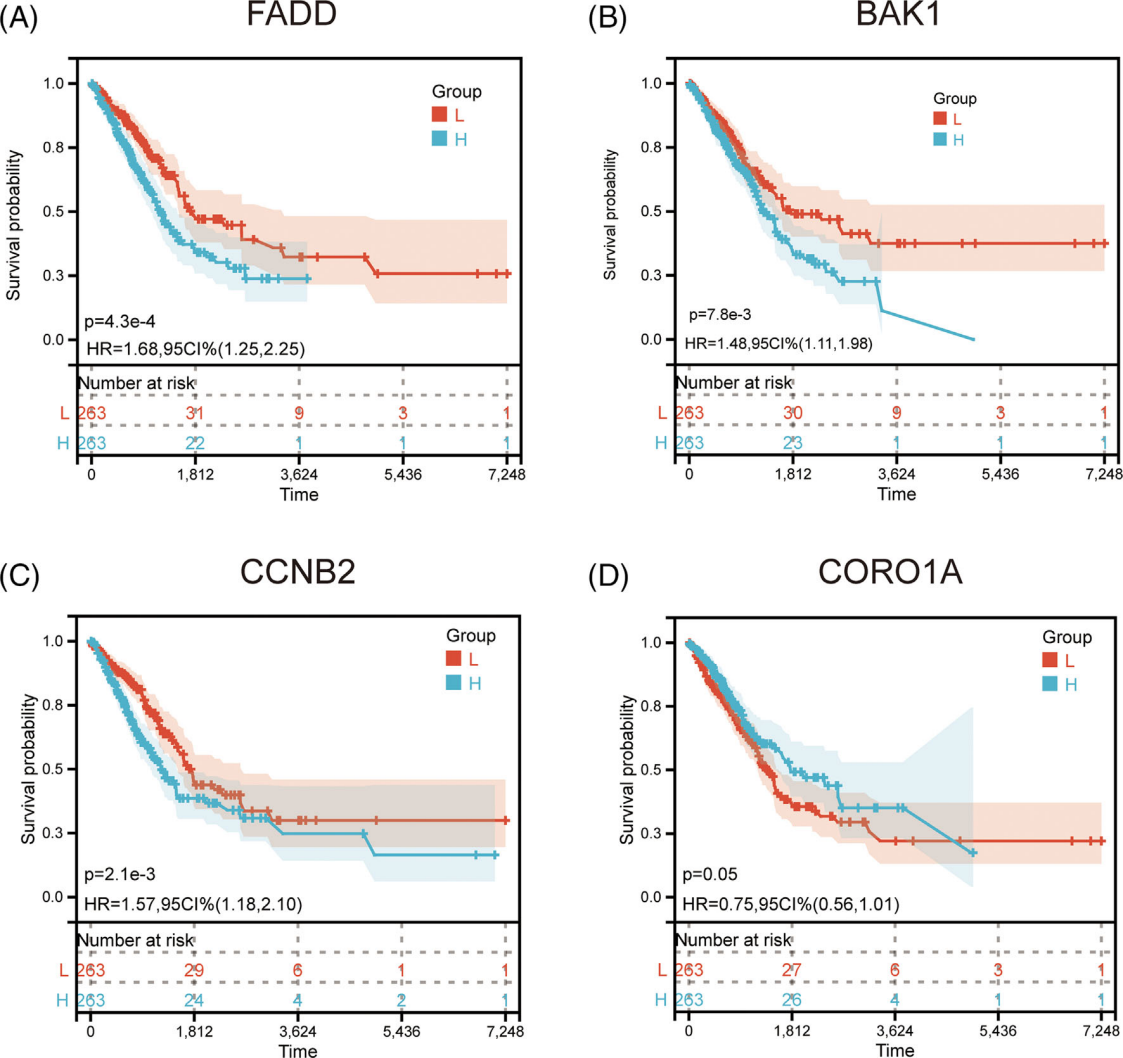
Survival analysis of risk model genes. (A–C) The prognosis of FADD, BAK1, and CCNB2 in the high‐risk group was poor and (D) CORO1A had a poor prognosis in the low‐risk group.

**FIGURE 4:**
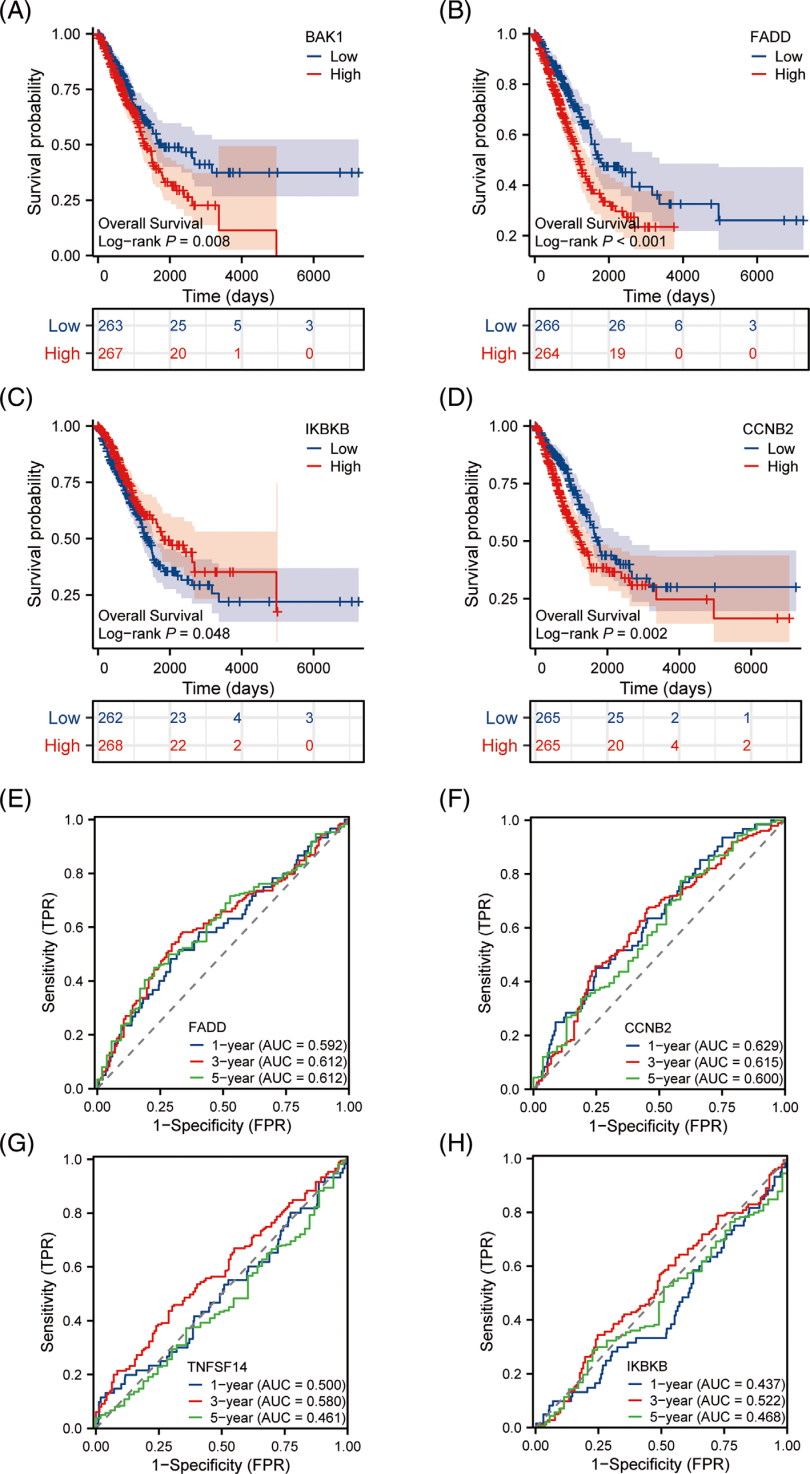
Survival analysis of risk factors for predictive models. (A–D) Survival analysis with statistically significant prediction models of risk factors and (E–H) ROC verified the differential efficacy of the predictive risk factors for overall survival outcomes in 1‐, 3‐, and 5‐year LUAD patients.

Finally, we analyzed the OS prognosis of risk model. We analyzed the OS prognosis of risk model. The statistically significant survival analysis showed that FADD, BAK1, and CCNB2 were highly expressed in the high‐risk group and the prognosis was poor (Figure 3A–C). CORO1A was highly expressed in the low‐risk group and had a poor prognosis (Figure 3D). Survival curves grouped by median gene expression suggested that patients with high CCNB2 (*p* = 0.002), FADD (*p* < 0.001), and BAK1(*p* = 0.008) expression had a significantly poorer OS prognosis (Figure 4A,B,D). And patients with low IKBKB expression (*p* = 0.0048) had significantly worse OS outcomes (Figure 4C).

### 3.3 OS prediction efficacy of risk factors in the risk model

The ROC curves with statistical significance at 1, 3, and 5 years suggested that TNFSF14 among the risk factors in the prediction model had significantly different OS outcomes for LUAD patients, including FADD (1 [AUC = 0.592], 3 [AUC = 0.612], and 5 years [AUC = 0.612]) (Figure 4E), CCNB2 (1 [AUC = 0.629], 3 [AUC = 0.615], and 5 years [AUC = 0.600]) (Figure 4F), BAK1 (*p* = 0.008), TNFSF14 (1 [AUC = 0.500], 3 [AUC = 0.580], and 5 years [AUC = 0.461]) (Figure 4G), IKBKB (1 [AUC = 0.437], 3 [AUC = 0.522], and 5 years [AUC = 0.468]) (Figure 4H).

### 3.4 Enrichment analysis

**FIGURE 5:**
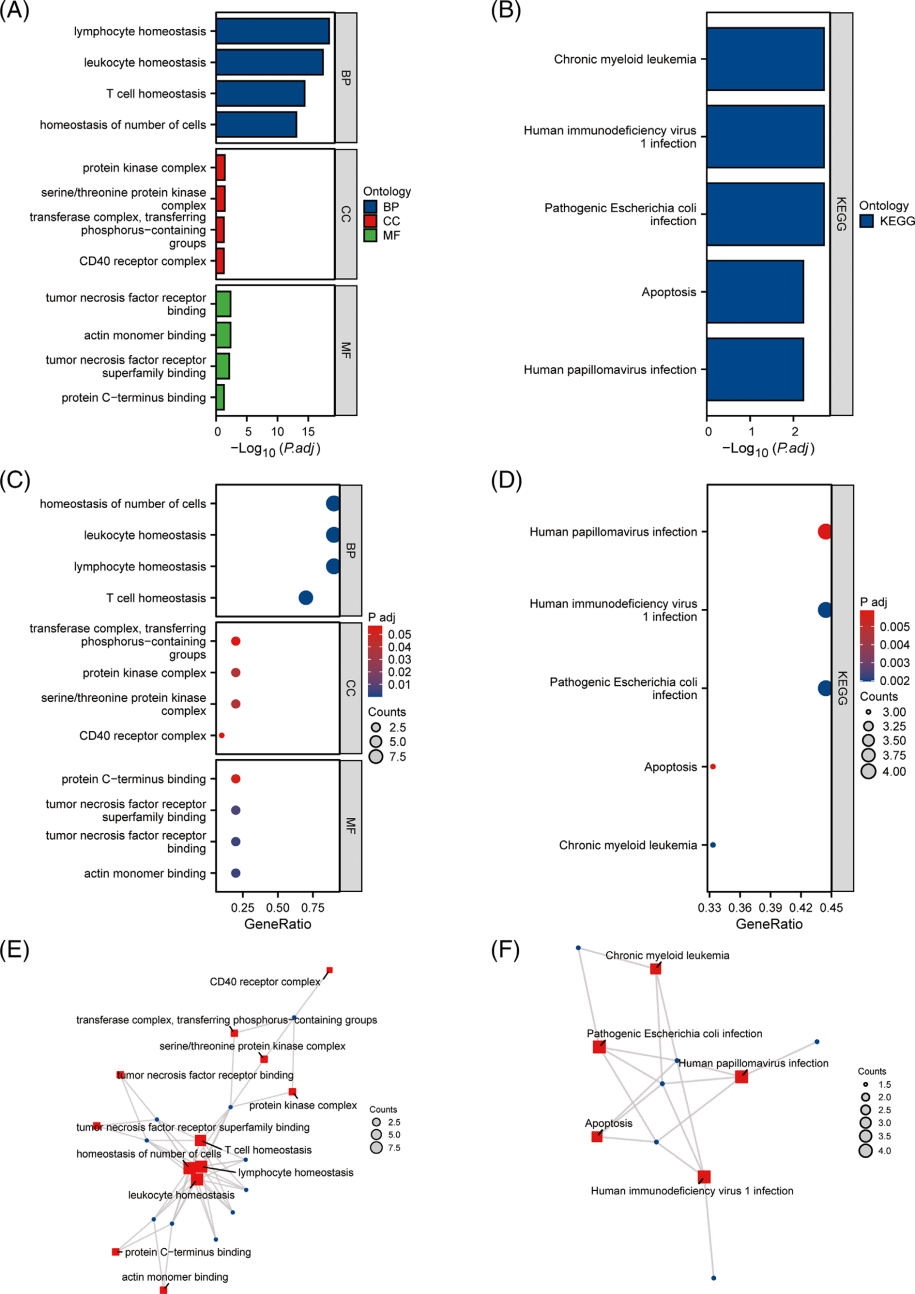
Enrichment analysis of immune cell homeostasis‐related prognostic genes. (A) Bar plot with column length representing the number of gene enrichment of GO; (B) bubble plot representing the number of gene enrichment and color representing significance of KEGG, with significance increasing gradually from yellow to blue; (C) bar plot with column length representing the number of gene enrichment of GO; (D) bubble plot representing the number of gene enrichment and color representing significance of KEGG, with gradual increase from yellow to blue; and (E,F) GO and KEGG Enrichment Analysis Enrichment Network diagram.

We performed enrichment analysis of a 10‐gene risk model for the prognosis of OS in LUAD patients. GO results showed that biological processes were enriched in lymphocyte homeostasis, leukocyte homeostasis, T cell homeostasis, homeostasis of number of cells, and a collection of T cell proliferation. Cell components were enriched in serine/threonine protein kinase complex, protein kinase complex, CD40 receptor complex, transferase complex, transferring phosphorus‐containing groups, and integral component of nuclear inner membrane. Molecular functions were enriched in actin monomer binding, tumor necrosis factor receptor binding, tumor necrosis factor receptor superfamily, protein C‐terminus binding, and actin filament binding (Figure 5A,C,E). Pathway enrichment analysis of the KEGG database suggests that the prognostic risk model is significantly associated with pathogenic Escherichia coli infection, human immunodeficiency virus infection, chronic myeloid leukemia, human papillomavirus infection, and apoptosis pathways (Figure 5B,D,F). The results are presented in bar, bubble, and chord plots.

### 3.5 Immune infiltration analysis of prognostic risk model

**FIGURE 6:**
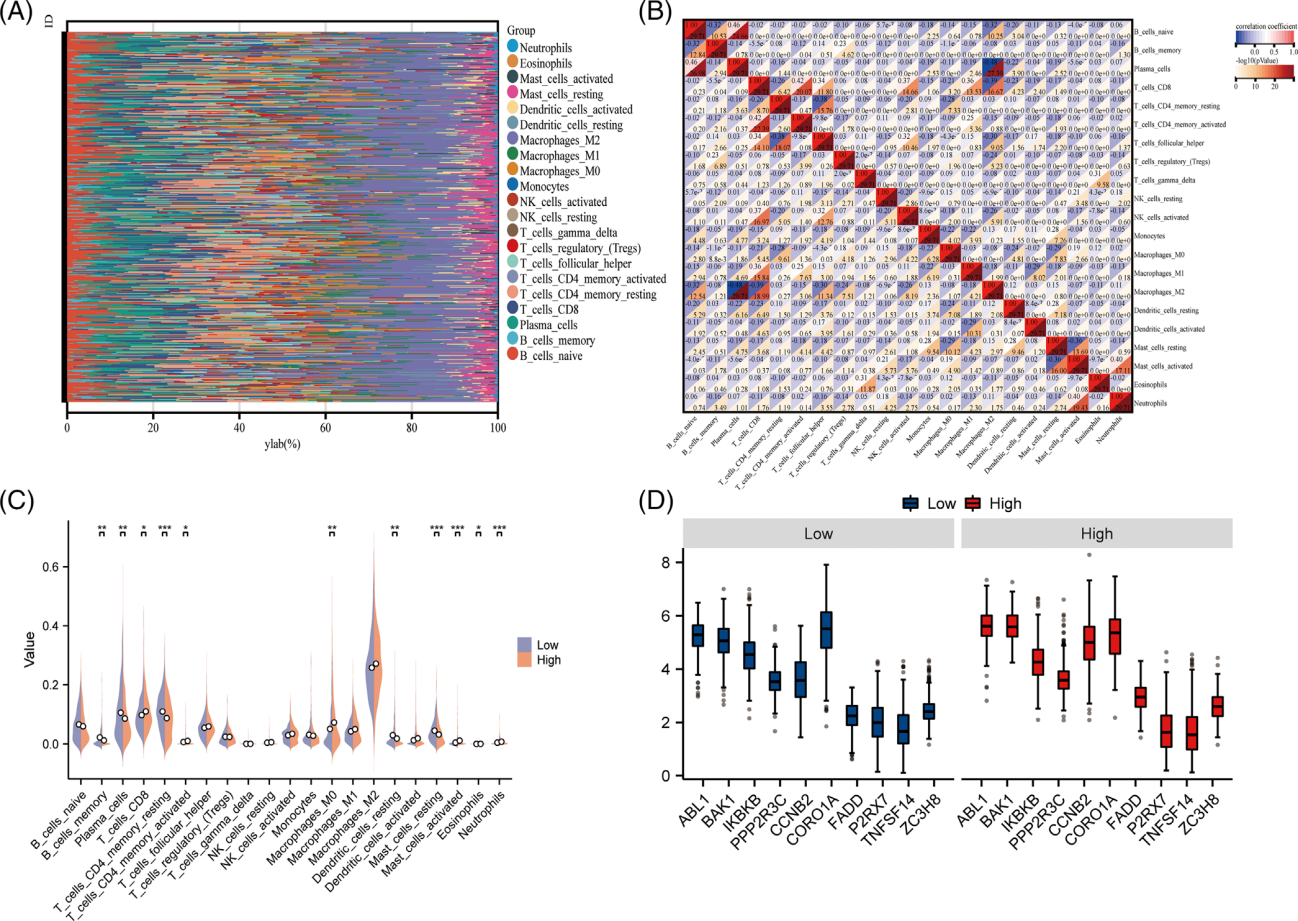
Evaluation of immune infiltration. (A) Stacked bar graphs showing infiltrating immune cells; (B) correlation heatmap of infiltrating immune cell distribution, horizontal axis indicates the type of immune cells; (C) violin plot of differences in immune cell infiltration (Wilcoxon test *<0.05; ^***^<0.001); and (D) box diagram of different cell infiltration in immune microenvironment of risk population.

Based on the CIBERSORT algorithm, we calculated the proportion of 22 immune cells in each LUAD sample and showed the proportion of 22 immune cells infiltrated in the tissue samples of LUAD patients by stacking bar charts (Figure 6A), while most of the immune cells showed significant negative correlation (Figure 6B). The ratio of low‐risk group was significantly higher. Found B cell memory, plasma cells, T cell CD4 memory resting, dendritic cell resting, and mast cell resting were significantly less infiltrated in the high‐risk group. We not only analyzed the differences in the infiltration of various immune cells between the high‐ and low‐risk groups (Figure 6C) but also compared the differences in the infiltration of different cells in the immune microenvironment between the high‐ and low‐risk groups (Figure 6D).

**FIGURE 7:**
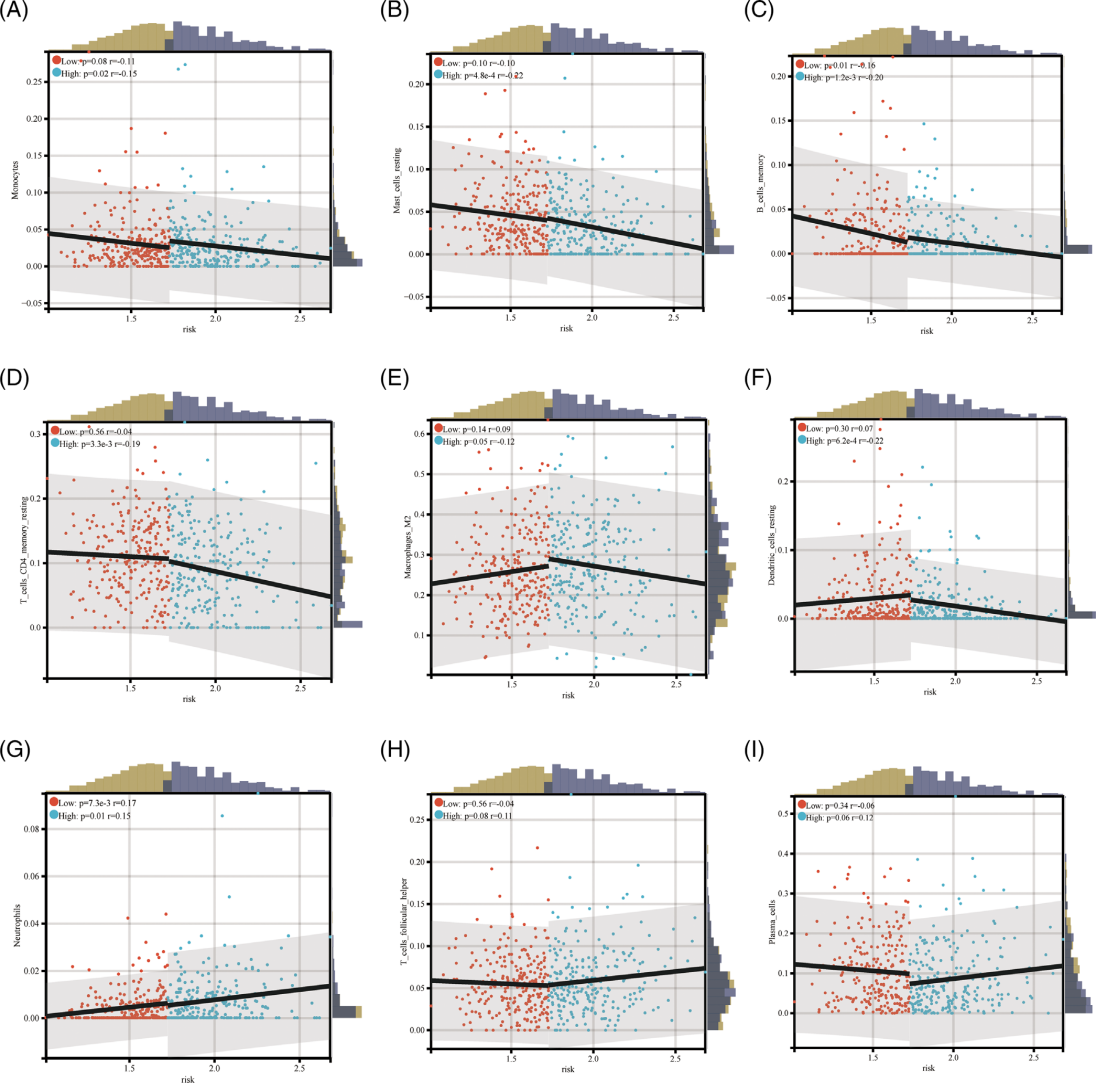
Correlation of immune cell homeostasis prognostic model risk scores. (A–F) Risk scores in patients at high risk for LUAD were significantly negatively correlated with immune cell infiltration and (G–I) risk scores in high‐risk patients with LUAD were significantly positively correlated with immune cell infiltration.

In our results, Figure 7 presents a scatter plot of the association between immune cell infiltration and a significant inverse association with risk scores in high‐risk LUAD patients: monocytes (low: *p* = 0.08, *r* = −0.11, high: *p* = 0.02, *r* = −0.15) (Figure 7A), mast cell resting (low: *p* = 0.10, *r* = −0.10, high: *p* = 4.8e‐4, *r* = −0.22) (Figure 7B), B cell memory (low: *p* = 0.01, *r* = −0.16, high: *p* = 1.2e‐3, *r* = −0.20) (Figure 7C), T cell CD4 memory resting (low: *p* = 0.56, *r* = −0.04, high: *p* = 3.3e‐3, *r* = −0.19) (Figure 7D), macrophage M2 (low: *p* = 0.14, *r* = 0.09, high: *p* = 0.05, *r* = −0.12) (Figure 7E), and dendritic cell resting (low: *p* = 0.30, *r* = 0.07, high: *p* = 6.2e‐4, *r* = −0.22) (Figure 7F). Figure 7G–I shows a scatter plot of the correlation between immune cell infiltration and risk score in high‐risk LUAD patients with significant positive correlations: neutrophils (low: *p* = 7.3e‐3, *r* = 0.17, high: *p* = 0.01, *r* = 0.15) (Figure 7G), T cell follicular helper (low: *p* = 0.56, *r* = −0.04, high: *p* = 0.08, *r* = 0.11) (Figure 7H), and plasma cells (low: *p* = 0.34, *r* = −0.06, high: *p* = 0.06, *r* = 0.12) (Figure 7I). The degree of infiltration of other immune cells was not significantly correlated with the risk score and is therefore not shown.

### 3.6 Experimental validation of the reliability of bioinformatics models

**FIGURE 8:**
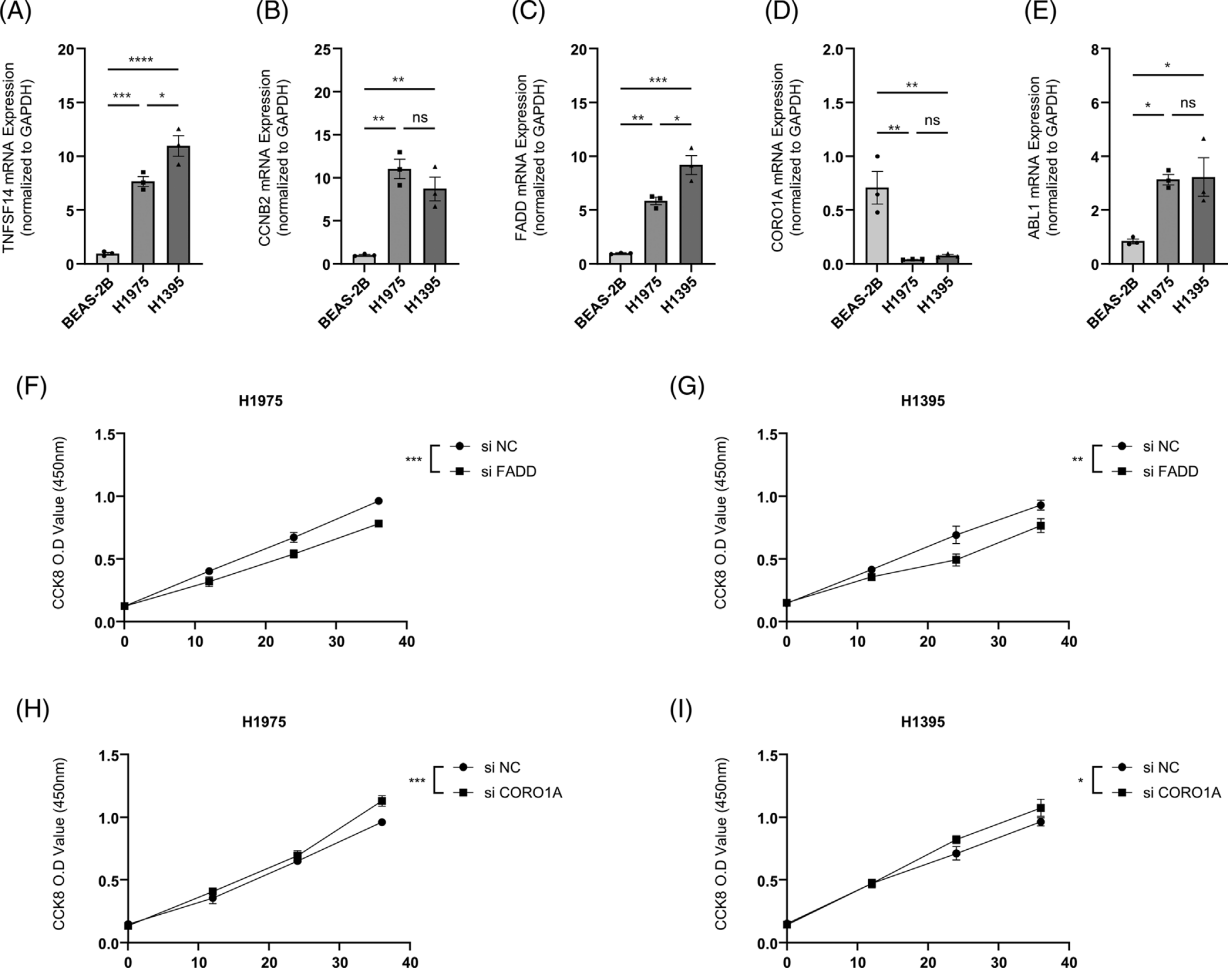
Validating the reliability of bioinformatics models. (A–E) PCR assay to detect mRNA expression of TNFSF14, CCNB2, FADD, CORO1A, and ABL1 in BEAS‐2B, H1975, and H1395 cell lines; (F,G) alterations in cell viability following inhibition of FADD expression in H1975 and H1395 cell lines; and (H,I) alterations in cell viability following inhibition of CORO1A expression in H1975 and H1395 cell lines. *N* = 3, ns > 0.05, *≤0.05, ^**^≤0.01, ^***^≤0.001, ^****^≤0.0001. The results are presented as mean ± SEM.

To validate the reliability of models predicted by bioinformatics, we examined mRNA expression of TNFSF14, CCNB2, FADD, CORO1A, and ABL1 by PCR in BEAS‐2B, H1975, and H1395 cell lines. PCR results showed that TNFSF14, CCNB2, FADD, and ABL1 were significantly increased in LUAD cell lines H1975 and H1395 (Figure 8A–C,E), while CORO1A expression was decreased in LUAD cell lines (Figure 8D). Subsequently, in H1975 and H1395 cell lines, we inhibited the expression of FADD or CORO1A. The results showed a decrease in cell activity after inhibition of FADD expression in LUAD cell lines (Figure 8F,G). In contrast, cell activity increased after inhibition of CORO1A expression in LUAD cell lines (Figure 8H,I). These findings indicate the reliability of the bioinformatics prediction model.

## 4 DISCUSSION

In our study, we constructed a novel survival risk prognostic model for maintaining immune homeostasis characteristics and validated gene expression in clinical samples. However, the underlying mechanism of how these genes regulate the LUAD process in our prognostic traits remains unclear. Therefore, immune cell homeostasis is an emerging field of cancer research. At the same time, the genes identified in this study are important for updating risk prognostic features in LUAD.

According to LASSO coefficients of 41 immune cell homeostasis‐related genes, a prognostic risk score model based on OS in LUAD was established. Lambda and min values are visualized using the LASSO logit model algorithm. Ten prognostic risk factors were included in multivariate Cox regression model, and five prognostic regulatory factors were found by forest map. TNFSF14, CCNB2, FADD, and ABL1 were risk factors, while CORO1A was protective factor for LUAD. Combined with the survival prognostic significance of 10 genes in high‐risk group and high‐expression group, it was found that the time‐dependent ROC curve also supported their prognostic significance. As well as the ROC curve at 1, 3, and 5 years suggested that the prognostic risk model had significant prognostic significance and could effectively identify the OS.

Numerous research results suggest the clinical significance of FADD as a therapeutic target for cancers.^18^, ^19^ FADD was initially described as a connector molecule for death receptor‐mediated apoptosis, and subsequent studies have demonstrated its antitumorigenicity and possibly tumorigenicity.^20^, ^21^ It is closely related to various immune and metabolic pathways.^22^ The expression of TNFSF14 in hepatocellular carcinoma (HCC) and other tumor tissues is significantly lower, and the HCC patients with low expression of TNFSF14 have high malignancy degree and poor prognosis.^23^ According to relevant studies, TNFSF14 is associated with mucosal immune dysregulation.^11^ What is more, BCR‐ABL1 has become the gold standard for monitoring patient prognosis.^13^, ^24^, ^25^ The genomic background of ABL1 is highly heterogeneous in tumor tissue but has not been well defined.^26^ IKBKE is an atypical inflammatory kinase that is a key regulator of the immune system, inflammation, and cancer.^27^, ^28^ Often amplified or activated, it plays a major carcinogenic role in human cancers.^29^, ^30^ Recent studies have used P2RX7 as a presumed target for gastric cancer, described its potential association with poor prognosis of gastric cancer, and clarified that P2RX7 in the growth and metastasis of gastric cancer.^31^, ^32^ Other studies have shown that P2RX7 is potentially related to the immune mechanism of inflammation and adhesion, which is of research value.^33^

Infiltration and aggregation of immune cell subsets affect immune homeostasis.^34^ However, the prognostic value of genes involved in the maintenance of immune homeostasis has not been well studied. The accumulation of immune cells and the infiltration of immune cell subsets are closely related to the maintenance of immune homeostasis.^35^ However, the prognostic value involved in the maintenance of immune homeostasis has not been well studied. Negative feedback regulation of immunohomeostasis maintenance plays an important role in tumor.^9^

CIBERSORT deconvolution was used to calculate the infiltration of immune cells. We not only analyzed the differences in various immune cells but also compared the differences in the infiltration of different cells in the immune microenvironment between different risk groups. The population with high degree of immune cell invasion has a lower prognosis risk, especially macrophage M2 and dendritic cells in our analysis. Particularly, the risk score is positively correlated with macrophage M2 and dendritic cell infiltration in the low‐risk group, while negatively correlated with high‐risk group, which more prominently indicated that the risk model constructed potential mechanism of regulation with the immune microenvironment of LUAD. Based on the correlation of immune cell infiltration between different risk groups, the risk score was associated with a significant inverse correlation, meaning that patients with higher risk had less immune cell infiltration in the internal environment, especially in monocytes, mast cell resting, B cell memory, T cell CD4 memory resting, macrophage M2, and dendritic cell resting. The comprehensive results of immune cell infiltration analysis more prominently illustrate that the risk model we constructed is closely related to LUAD microenvironment.

We examined the differences in the expression of the 10 gene models constructed and their effects on cell viability at the cellular level. The results showed that the mRNA expressions of TNFSF14, CCNB2, FADD, CORO1A, and ABL1 in BEAS‐2B, H1975, and H1395 cells were significantly different by PCR. At the same time, inhibition of FADD and CORO1A expression in H1975 and H1395 cell lines significantly increased cell viability. These experimental results support that the molecular model we constructed has significant biological significance and potential application value in lung cancer prognosis. Despite the presence of a large number of tumor markers in LUAD, studies based on genes associated with maintaining immune homeostasis have not been reported. In order to reveal the molecular mechanism of the effect of this immune homeostasis risk model on the prognosis and survival of patients with LUAD, subsequent biological and cellular experiments are needed.

To investigate the impact of genetic models on the immune microenvironment, we compared the relationship between the immune homeostasis risk score and the extent of immune cell infiltration and found that the risk score was able to delineate the infiltration status of the majority of immune cells.^28^, ^29^ Thus, low‐risk LUAD patients identified on the basis of features of immune homeostasis should have a better prognosis and can be used as an independent prognostic factor. However, the study has some limitations. First, as a retrospective dataset, prospective analysis based on immune homeostasis characteristics is required.^36^ Second, all expression data are derived from public datasets, and the results must be confirmed using new methods and fresh specimens.^30^ Third, we assessed the potential to indirectly predict OS outcomes in LUAD, and more studies are needed to confirm this conclusion.

In summary, we identified 10 hub prognostic genes associated with maintaining immune homeostasis in LUAD. Prognostic markers showed good accuracy, and it was inferred that they could be used as promising therapeutic targets. These immunohomeostasis related prognostic risk genes mediate LUAD progression and contribute to poor prognostic functions that require further clinical investigation.

## AUTHOR CONTRIBUTIONS

Yidan Sun and Yixun Chen contributed equally to this work.

## CONFLICT OF INTEREST STATEMENT

All authors declare no competing nonfinancial/financial interests.

## ETHICS STATEMENT

The experimental protocol was established, according to the ethical guidelines of the Helsinki Declaration. Ethical permissions were granted by the Human Ethics Committee at Affiliated Hospital of Nantong University (No. 2018‐K020).

## CONSENT

Written informed consent was obtained from individual or guardian participants included in this study.

## Supporting information


**Figure S1.** The expression of immune homeostasis prognostic model gene was significantly different in LUAD samples.

## Data Availability

The data that support the findings of this study are openly available in The Cancer Genome Atlas (TCGA) at https://portal.gdc.cancer.gov/.
