# Supplementary material for: Identification and analysis of prognostic immune cell homeostasis characteristics in lung adenocarcinoma
Source: Clin Respir J. 2024 May 17;18(5):e13755. doi: 10.1111/crj.13755 (PMC11099951; doi:10.1111/crj.13755)

Supplementary Fig.1 The expression of immune homeostasis prognostic model gene was significantly different in LUAD samples.


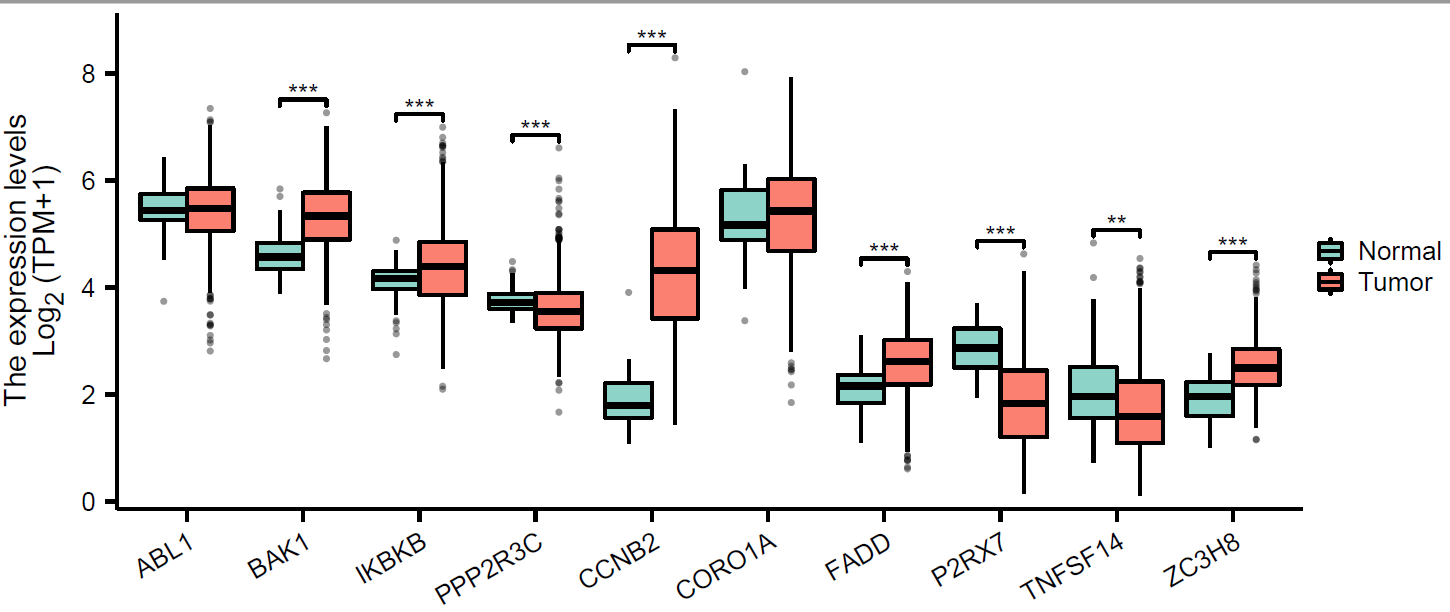

Supplement: Supplementary file 1 — Figure S1. The expression of immune homeostasis prognostic model gene was significantly different in LUAD samples. [file CRJ-18-e13755-s001.docx]
